# Top-Down Proteomics of Mouse Islets With Beta Cell CPE Deletion Reveals Molecular Details in Prohormone Processing

**DOI:** 10.1210/endocr/bqad160

**Published:** 2023-11-15

**Authors:** James M Fulcher, Adam C Swensen, Yi-Chun Chen, C Bruce Verchere, Vladislav A Petyuk, Wei-Jun Qian

**Affiliations:** Environmental Molecular Sciences Laboratory, Pacific Northwest National Laboratory, Richland, WA 99354, USA; Integrative Omics, Biological Sciences Division, Pacific Northwest National Laboratory, Richland, WA 99354, USA; Department of Surgery, BC Children’s Hospital Research Institute and University of British Columbia, Vancouver, British Columbia, V5Z 4H4, Canada; Department of Surgery, BC Children’s Hospital Research Institute and University of British Columbia, Vancouver, British Columbia, V5Z 4H4, Canada; Department of Pathology and Laboratory Medicine, BC Children’s Hospital Research Institute and University of British Columbia, Vancouver, British Columbia, V5Z 4H4, Canada; Integrative Omics, Biological Sciences Division, Pacific Northwest National Laboratory, Richland, WA 99354, USA; Integrative Omics, Biological Sciences Division, Pacific Northwest National Laboratory, Richland, WA 99354, USA

**Keywords:** pancreas, diabetes, IAPP, insulin, CPE, islets, proinsulin, proteoforms

## Abstract

Altered prohormone processing, such as with proinsulin and pro-islet amyloid polypeptide (proIAPP), has been reported as an important feature of prediabetes and diabetes. Proinsulin processing includes removal of several C-terminal basic amino acids and is performed principally by the exopeptidase carboxypeptidase E (CPE), and mutations in CPE or other prohormone convertase enzymes (PC1/3 and PC2) result in hyperproinsulinemia. A comprehensive characterization of the forms and quantities of improperly processed insulin and other hormone products following *Cpe* deletion in pancreatic islets has yet to be attempted. In the present study we applied top-down proteomics to globally evaluate the numerous proteoforms of hormone processing intermediates in a β-cell-specific *Cpe* knockout mouse model. Increases in dibasic residue–containing proinsulin and other novel proteoforms of improperly processed proinsulin were found, and we could classify several processed proteoforms as novel substrates of CPE. Interestingly, some other known substrates of CPE remained unaffected despite its deletion, implying that paralogous processing enzymes such as carboxypeptidase D (CPD) can compensate for CPE loss and maintain near normal levels of hormone processing. In summary, our quantitative results from top-down proteomics of islets provide unique insights into the complexity of hormone processing products and the regulatory mechanisms.

Islet prohormone processing, including proinsulin, pro-islet amyloid polypeptide (proIAPP), and proglucagon processing, has been reported to be altered during both prediabetes and diabetes (1, 2). In the case of type 1 diabetes (T1D), dysregulation of prohormone processing (3, 4), including the carboxypeptidase E (CPE) pathway (2, 5), has been extensively reported. Emerging data have suggested that β-cell dysfunction may exacerbate the development and progression of T1D (4) and evidence of increased proinsulin or proIAPP relative to mature insulin or IAPP expression has been reported either in circulation or in the islet at various stages in T1D (3, 6-9). Central to the processing of these hormones is CPE, and multiple studies have shown the significance of CPE in the prohormone processing pathways (10, 11), its necessary colocalization (12, 13), and enzymatic characteristics and regulation (14‐16). Notably, while β-cell CPE deficiency does not result in spontaneous onset of obesity and hyperglycemia (17), it leads to accelerated development of streptozotocin-induced hyperglycemia in mice, pointing toward protective roles of CPE in the progression of T1D. A detailed molecular-level characterization of all proteins and hormones impacted by CPE enzyme dysfunction in pancreatic islets has yet to be attempted but would be of considerable importance for understanding the molecular underpinnings of individuals with dysfunctional insulin processing.

We therefore sought to study the impact of CPE enzyme dysfunction on prohormone processing by utilizing a β-cell specific *Cpe* knockout (KO) mouse model (β*Cpe*KO; *Cpe*^fl/fl^*×Ins1*^Cre/+^*)* (17) in combination with top-down proteomics (18). Top-down proteomics analyzes proteins in their intact state (ie, not enzymatically digested into peptides as in traditional proteomics) through mass spectrometry. These intact protein species are typically referred to as “proteoforms,” a term encompassing amino acid mutations, alternative splicing events, proteolytic cleavages, and other posttranslational modifications. We found significant alterations in protein processing in the islets from β*Cpe*KO mice, with changes in proteoform quantities extending beyond the anticipated hormones with known CPE processing. Both full-length unprocessed and truncated insulin proteoforms were quantified, along with a total of 468 murine insulin (INS1 and INS2) proteoforms. Other CPE-processed hormones were also affected in similar patterns (notably: IAPP, SCG2, CHGA, SST, and CHGB), as well as additional proteins that have not been described as interacting directly with CPE (notably: HMGN3, TPT1, and TCEAL3). Interestingly, several known CPE substrates (eg, mature IAPP) were not significantly affected by *Cpe* knockout, indicating potential compensatory processing by paralogous processing enzymes, such as CPD. Taken together, these findings will enable better understanding of one of the underlying pathways of dysfunction present in β cells prior to disease onset, particularly in the context of CPE and insulin processing. The proteoforms we identified will also be useful toward developing prognostic/diagnostic methods in at-risk individuals or investigating targets for therapeutic interventions.

## Methods

### Sample Preparation

Mice were generated from crossing male *Ins1*^Cre/+^  *Cpe*^fl/fl^ and female *Cpe*^fl/fl^ mice, and littermates were used. Islets from the wild-type (WT) and βCpeKO mice were isolated following previous protocol (19). All studies were approved by the Animal Care and Use Committee at the University of British Columbia. Approximately 100 islets were hand-picked and transferred to a 1.5-mL Eppendorf tube before being frozen at −80 °C. Immediately before lysis of islets, samples were transferred onto ice. To release cell contents and denature proteins, 466 µL of homogenization buffer (8 M urea, 100 mM ammonium bicarbonate [ABC], 5 mM EDTA, 1 mM PMSF) was added to each sample before vortexing for 10 seconds to resuspend islets followed by sonication in a water bath for 5 minutes at room temperature. Reduction was accomplished through addition of 14 µL of 0.5 M TCEP with incubation at room temperature for 2 hours within a ThermoMixer (ThermoFisher) set at 1200 RPM. This was followed by alkylation using 20 µL of 0.5 M iodoacetamide (IAA) and incubation for 30 minutes at room temperature in complete darkness at 1200 RPM. The reaction was quenched through addition of 50 µL of dithiothreitol (DTT) before clarification of samples with 15 minutes of centrifugation at 18 000 RCF at 10 °C. The resulting supernatant was added to a 3 K MWCO Amicon® Ultra 0.5 mL centrifugal filter (MilliporeSigma) and centrifuged at 14 000 RCF for 60 minutes at 10 °C. Then ∼50 to 70 µL of retentate was then diluted with 0.5 mL wash buffer (8 M urea, 10 mM ABC, 2 mM EDTA) followed by centrifugation at 14 000 RCF for 60 minutes at 10 °C. This step was performed once more to ensure >100-fold dilution of reducing and alkylating compounds.

Retentates were then transferred to a 1.5-mL LoBind Eppendorf tube (Eppendorf) and diluted 1:1 (v/v) with 1% formic acid in milliQ H_2_O before concentration determination with a bicinchoninic acid assay. For the bicinchoninic acid assay, bovine serum albumin (BSA) calibration standards were prepared in the same solution as the samples (4 M urea, 5 mM ABC, 1 mM EDTA, 0.5% formic acid [FA]) to account for any buffer interference, and duplicate measurements were performed for better estimation of concentration. Prior to liquid chromatography–mass spectrometry (LC-MS) analysis, samples were adjusted to equivalent concentrations (0.04 mg/mL) with 4 M urea, 5 mM ABC, 1 mM EDTA, 0.5% FA. To reduce any potential for sample loss due to nonspecific binding to surfaces, samples were added to polypropylene PCR tubes inserted into LC-MS vials (20). It should be noted that to prevent endogenous CPE protease activity during sample preparation, tissue homogenates were kept in at least 4M urea throughout the entire process.

### LC-MS/MS Analysis

Samples were analyzed using a Waters NanoACQUITY UPLC system with mobile phases consisting of 0.2% FA in H_2_O (Mobile Phase A) and 0.2% FA in acetonitrile (ACN) (Mobile Phase B). Both trapping-precolumn (150 µm i.d., 5-cm length) and analytical column (100 µm i.d., 50-cm length) were slurry-packed with C2 packing material (5 µm and 3 µm for trap/analytical respectively, 300 Å, Separation Methods Technology). Samples were loaded into a 10-µL loop, corresponding to 400 ng of loaded material, and injected into the trapping column with an isocratic flow of 1% B at 5 µL/min over 15 minutes for desalting. Separation was performed with a 1% to 50% B gradient over 140 minutes at 300 nL/min. For MS/MS analysis of proteins, the NanoACQUITY system was coupled to a Thermo Scientific Orbitrap Fusion™ Lumos™ Tribrid™ mass spectrometer equipped with the FAIMS Pro interface. Source parameters included electrospray voltage of 2.2 kV, transfer capillary temperature of 275 °C, and ion funnel RF amplitude of 30%. To increase proteome coverage, we utilized an internal compensation voltage (CV) stepping approach with high-field asymmetric ion mobility (FAIMS). Based on prior work that has demonstrated the ideal CV range for increasing identifications, we utilized 3 FAIMS CVs at −55 V, −45 V, and −35 V (21-23). FAIMS was set to standard resolution without supplementary user-controlled carrier gas flow and a dispersion voltage (DV) of −5 kV (equivalent to a dispersion field of −33.3 kV/cm) (24), while the CV switched between 3 voltages (−55, −45, and −35) throughout data collection (referred to as *internal CV stepping*) (25).

The Fusion Lumos was set to “Peptide” acquisition mode, and data were collected as full profile. MS^1^ and MS^2^ data were acquired at a resolution of 120k and 60k, 2 microscans across a 500 to 2000 *m/z* range, and with Automatic Gain Control (AGC) targets of 1E6 and 5E5, respectively. MS^1^ and MS^2^ were acquired with a maximum inject time of 250 ms. Data dependent settings included selection of top 6 most intense ions, exclusion of ions lower than charge state 3+, exclusion of undetermined charge states, and dynamic exclusion after 1 observation for 30 seconds. Ions selected for MS^2^ were isolated over a ±1.5 *m/z* window and fragmented through collision-induced dissociation (CID) with a normalized collision energy of 35%.

### Data Analysis

Proteoform identification was performed with TopPIC version 1.4 (26). Settings for TopPIC included a precursor window of 3 *m/z* (to account for isotopic envelope), mass error tolerance of 15 ppm, a proteoform cluster error tolerance of 0.8 Da, a mass shift upper bound of 275 Da and lower bound of −150 Da, and a maximum number of allowed unknown modifications of 1. MS^2^ spectra were searched against the most recent Swiss-Prot database for *Mus musculus* containing 17 058 entries, a splice variant database containing 8281 splice-isoform entries, and a TrEMBL database containing 38 416 entries (UP000000589 - accessed July 1, 2021). All databases were scrambled to generate decoys. A list of 12 dynamic modifications were provided during the open modification search in order to reduce the number of unknown mass shifts. Proteoform spectrum matches were filtered to achieve a 1% false discovery rate (FDR). Downstream data analysis and figure generation was performed in the R environment. To perform label-free quantitation (LFQ), we utilized the R package TopPICR (27). Normalization (through median equalization), calculation of log_2_ fold-change, missing value percentage (ie, the proportion of missing values across samples for a given proteoform), *P* value, and adjusted *P* value (multiple testing corrected, FDR) were accomplished using the MSstats 4.0.1 R package (28). *P* values produced from the Student *t* test were adjusted for multiple tests using the Benjamini and Hochberg procedure. Unless otherwise noted, all *P* values referred to herein are adjusted *P* values. Note that we refer to proteoforms in the manuscript by gene, the starting/ending amino acid and their position relative to the full-length protein sequence. Posttranslational modifications are also notated in brackets alongside the amino acid that is modified.

## Results

### Evaluation of the Top-Down Proteomic Approach for Islet Profiling

**Figure 1.**
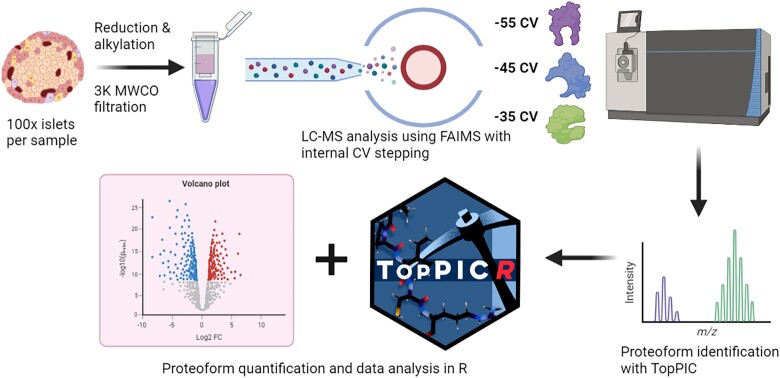
Workflow utilized for processing mouse islets for top-down proteomic analysis. Created with BioRender.com.

Islets isolated from 4 WT and 4 β*Cpe*KO mice were processed for top-down proteomic analysis following the method shown in Fig. 1. Prior to mass spectrometric analysis, it was noted that protein yields measured by bicinchoninic acid assay were found to be ∼3-fold higher for β*Cpe*KO islets compared to WT (Supplementary Table S1 (29)). This may be related to the increased β-cell area in βCpeKO islets (17). After LC-MS analysis and downstream data processing, 1636 proteoforms from 295 genes could be detected in total from the 8 datasets. Post-normalization, the distribution of relative standard deviations (RSDs) for the KO samples was within the expected ranges of technical variation based on previous top-down and bottom-up label-free quantitation (LFQ) studies (Supplementary Fig. S1 (29)) (30, 31). The RSD distribution for the WT group (2 male and 2 female) was modestly higher, which we believe may be attributable to biological differences related to sex, as the RSD distributions are lower when separated by sex (Supplementary Fig. S1 (29)). This suggests sex is a confounding variable and a limitation of our analysis; however, we should note that no proteoforms are statistically different in abundance between male and female mice. Therefore, impacts on quantification between WT and β*Cpe*KO should be limited.

**Figure 2.**
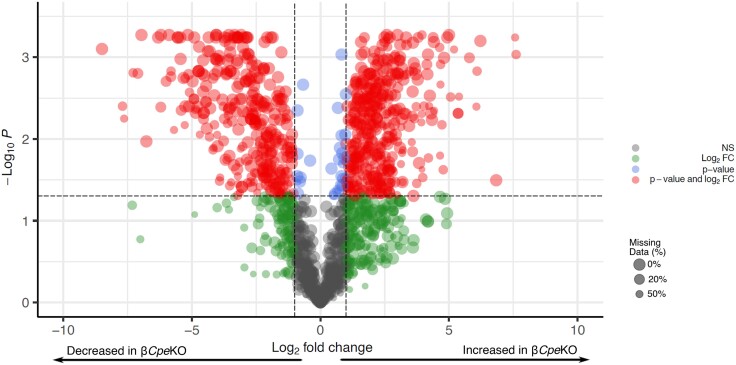
Volcano plot showing all proteoforms quantified. Horizontal dotted line indicates adjusted *P* value cutoff (.05) and vertical dotted lines indicate log_2_ fold-change cutoff of 1 and −1. Point size is scaled to the number of missing values present (ie, larger point size indicates fewer missing values for a given proteoform).

Of the 1636 proteoforms identified, 1369 could be quantified between the 2 conditions with the remainder only being observed in either condition. Using an adjusted *P* value cutoff of < .05 and log_2_ fold-change cutoff of ±1, numerous changes in proteoform abundance between β*Cpe*KO and WT mice could be revealed (Fig. 2). As demonstrated in Fig. 2, 777 proteoforms were found to have a significant log_2_ fold-change and adjusted *P* value. The remainder had either nonsignificant *P* values or log_2_ fold-changes. All MS^2^ spectrum matches identified and proteoforms quantified in this study are summarized in Supplementary Tables S2 and S3 (29), respectively.

### Proteoform Profiling Reveals Direct and Indirect Impacts of β*Cpe*KO

**Figure 3.**
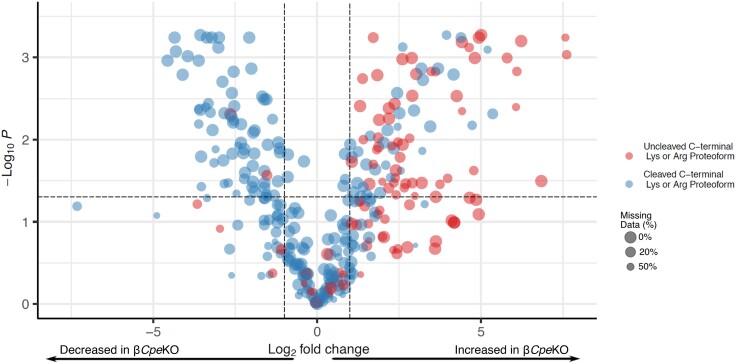
Volcano plot showing proteoforms that end on a basic residue or immediately preceding a basic residue and do not contain unknown modifications. Horizontal dotted line indicates adjusted *P* value cutoff (.05) and vertical dotted lines indicate log_2_ fold-change cutoff of 1 and −1. Point size is scaled to the number of missing values present.

Considering the role of CPE role as an exopeptidase that acts on proteins and peptides containing C-terminal basic residues (eg, Lys and Arg), proteoforms that are differentially abundant will likely fall into 2 categories: (i) proteoforms that are directly cleaved by CPE and (ii) proteoforms that are indirectly impacted by the change in abundances of CPE substrates. To investigate proteoforms that fall into the former category, we generated a volcano plot including each proteoform that contains a C-terminal basic amino acid or was cleaved just before a basic residue (Fig. 3). Many of these proteoforms (92 out of 249) remain unchanged and presumably represent a pool of proteoforms that are not authentic substrates of CPE or are compensated for with paralogous carboxypeptidases such as carboxypeptidase D (CPD).

For the proteoforms that have significant *P* values and log_2_ fold-changes, there is an unequal distribution relative to the presence or absence of a C-terminal basic amino acid. Of the proteoforms increasing in abundance, 62% (upper right quadrant, Fig. 3) have an uncleaved C-terminal basic amino acid (55 out of 63), while 96% of proteoforms decreasing in abundance (upper left quadrant, Fig. 3) are cleaved at the C-terminus before a basic residue (66 out of 68). We believe these proteoforms likely represent authentic CPE substrates based on the differential abundance and presence or absence of a C-terminal basic residue. Perhaps not surprisingly many could be attributed to INS1, INS2, SCG2, CHGA, SST, and CHGB*—*that is, known substrates of CPE (32, 33). However, to the best of our knowledge, the remainder have yet to be established as CPE substrates (a complete list of these potential substrates is provided in Supplementary Table S4 (29)).

**Figure 4.**
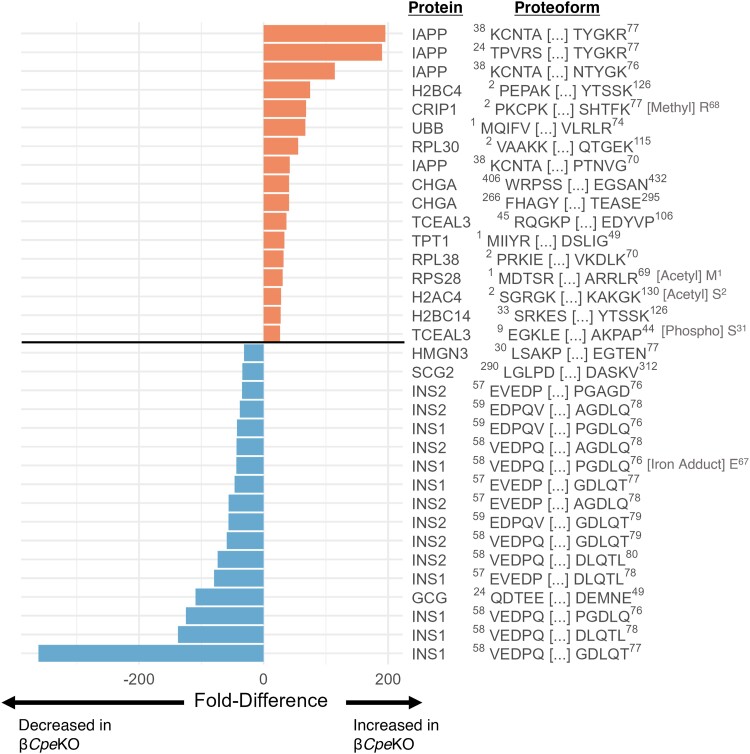
Bar chart showing the largest fold-changes of proteoforms in β*Cpe*KO islets. Proteoforms are displayed by gene and are shown with an abbreviated sequence indicating the starting and ending amino acid position, along with the first and last 5 amino acids as well as any posttranslational modifications as annotated. Proteoforms containing unknown modifications are excluded from this figure. All differentially abundant proteoforms presented were statistically significant with adjusted *P* values < .05.

We next investigated the proteoforms with the largest positive and negative fold-differences (Fig. 4). Those increasing in abundance typically have C-terminal basic residues while those decreasing in abundance are cleaved immediately before a C-terminal basic residue. Interestingly, many of those increasing have diverse biochemical functions and roles outside of what would be expected for substrates of CPE. Two proteoforms containing C-terminal basic residues are full-length histone proteins (H2BC4 ^2^P-K^126^ and H2AC4 ^2^S-K^130^ with fold-differences of 74.54 and 28 respectively). With H2BC4 ^2^P-K^126^, there is a corresponding proteoform also quantified that has a C-terminal basic residue cleaved with no change in abundance. This is a somewhat surprising observation, since one would expect proteoforms with C-terminal basic residues cleaved to have reduced abundance due to CPE deletion. Accordingly, we can see similar behavior with other histone proteins and several ribosomal subunit proteins, as well as a few other proteins with diverse functions (Table 1). These data suggest the presence of compensatory mechanisms for removal of carboxy basic residues. From this list of proteoforms, it is also notable that the EIF5A proteoform (^2^[Ac]A-[Hypusin]K^50^- K^154^) was identified as being hypusinated (Supplementary Fig. S2 (29)). POLR2M is an exception to the compensatory trends, as the corresponding proteoform with a cleaved C-terminal basic residue is 5.5-fold reduced in β*Cpe*KO, implicating CPE as the principal protease acting on this protein.

**Table 1.** Quantification of proteoforms that differ by the presence or absence of a C-terminal basic residue

Protein	Proteoforms	Uncleaved C-terminal basic residue (fold-difference)	Cleaved C-terminal basic residue (fold-difference)
POL2RM	^2^[Ac]A-**R**^84^	βCpeKO only	−5.50*^a^*
EIF5A	^2^[Ac]A-[Hypusin]K^50^- **K**^154^	18.76*^a^*	1.58
CRIP1	^2^P-[Methy]R^68^- **K**^154^	21.40*^a^*	1.22
H2BC4	^2^P-**K**^126^	74.54*^a^*	1.72
RPL38	^2^P-**K**^70^	32.00*^a^*	−1.26
RPS28	^1^[Ac]M-**R**^69^	30.48*^a^*	−1.59

^*a*^Indicates fold-change is statistically significant (adjusted *P* value < .05).

As mentioned above, the differentially abundant proteoforms observed within this study can be expected to either be direct substrates of CPE or indirectly impacted by the substantial changes related to CPE processing. There are several proteoforms within Fig. 4 that fall within the latter category, including HMGN3 ^30^L-N^77^ and TPT1 ^1^M-G^49^, which had a 31-fold decrease and 33-fold increase in abundance in β*Cpe*KO mice, respectively. With translationally controlled tumor protein (TPT1, also known as p23, fortilin, or histamine-releasing factor) we observed not only a ∼33-fold increase with TPT1 ^1^M-G^49^ but also a ∼7-fold increase of the longer TPT1 ^54^A-C^172^ proteoform, suggesting the intact protein may be considerably more abundant, or alternatively, is being proteolyzed into smaller inactive proteoforms (Supplementary Fig. S3 (29)). The protein TCEAL3 also presented 2 proteoforms that are both significantly increased in abundance (>16-fold) within β*Cpe*KO (Fig. 4 and Supplementary Fig. S4 (29)). Overall, the β*Cpe*KO phenotype reveals many yet-to-be-described proteoforms that show evidence of being direct substrates of CPE, as well as proteoforms that are indirectly impacted due to significant dysfunction in pancreatic islet hormone processing.

### Consequences of β*Cpe*KO on Canonical Pancreatic Hormones

**Figure 5.**
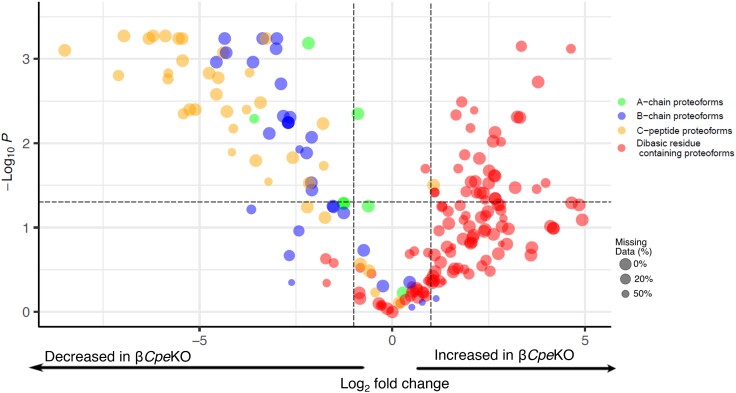
Volcano plot of INS1 and INS2 proteoforms quantified in β*Cpe*KO and WT islets. Proteoforms containing unknown modifications are excluded from this figure.

**Figure 6.**
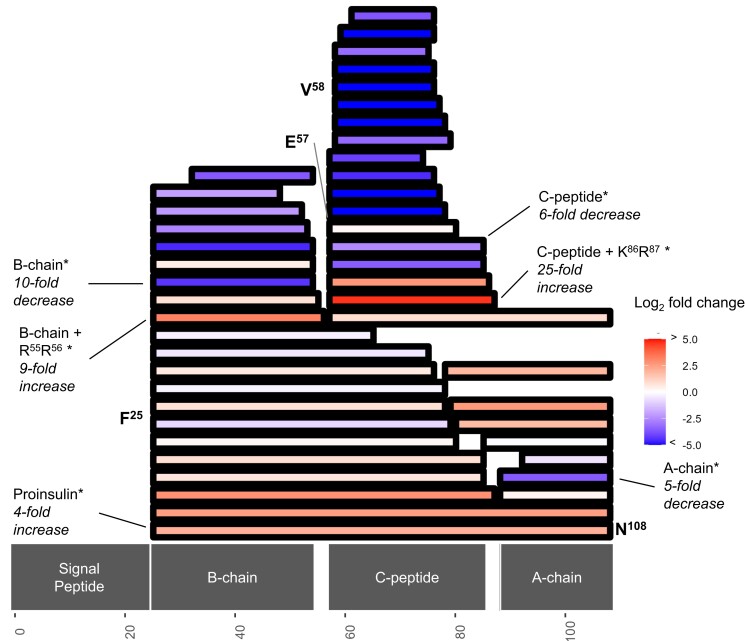
Mapping of INS1 proteoforms with statistically significant and insignificant (adjusted *P* value threshold of .05) changes in abundance (log_2_ fold-change threshold of ±1). Each rectangle indicates a unique proteoform. For proteoforms with text labels, * indicates statistically significant (adjusted *P* value < .05). Proteoforms containing unknown modifications are excluded from this figure.

As might be expected based on the role of CPE in hormone processing and the role of pancreatic islets themselves, the largest changes in abundance are related to INS1, INS2, and IAPP processing (Fig. 4; Supplementary Fig. S5 and S6 (29)). Globally quantifying INS1 and INS2 proteoforms reveals strikingly large increases in abundance of incompletely processed (ie, dibasic amino acid containing) insulin proteoforms in β*Cpe*KO relative to the WT pancreatic islets (Fig. 5). Many A-chain, B-chain, and C-peptide proteoforms are significantly reduced in abundance in β*Cpe*KO as well. By mapping the insulin proteoforms to the full-length insulin sequence, we can more directly visualize the impacts of β*Cpe*KO on insulin processing (INS1 in Fig. 6 and INS2 in Supplementary Fig. S7 (29)). Overall Ins1 and Ins2 show very similar differential abundance profiles, with proteoforms that are reduced in abundance mostly consisting of C-peptide and B-chain truncations while those increased in abundance are dibasic residue–containing. Indeed, as might be expected, intact proinsulin is 4 to 6-fold higher in abundance for INS1 and INS2 and the strongest decreases in abundance are linked to C-peptide truncations (Figs. 4 and 6).

However, there are a few apparent differences between INS1 and INS2. Specifically, INS1 demonstrates a 6-fold decrease in the canonical C-peptide sequence, while INS2 shows no significant change in C-peptide abundance. Furthermore, INS1 B-chain ending with the dibasic residues is 9-fold higher in abundance while the INS2 equivalent is not significantly different. It is also notable that both INS1 and INS2 appear to follow a C-terminal mediated degradation pathway that involves the sequential cleavage of proinsulin, B-chain, and C-peptide C-termini.

We also identified several novel proteoforms derived from the alternative splicing of the second intron of INS2 (Uniprot: D3Z596), including the recently discovered Disjointing peptide (D-peptide) which is thought to be C-terminally amidated by peptidylglycine α-amidating monooxygenase (PAM) (34). This alternative splicing event generates a frameshift mutation which introduces a partially distinct C-peptide sequence as well as an early stop-codon. Despite this significant difference in amino acid composition, D3Z596 proteoforms follow a similar pattern of processing and change in abundance as INS1 and INS2 in β*Cpe*KO (Supplementary Fig. S8 (29)). The longest D3Z596 proteoform (^25^F-G^79^) begins after the expected INS propeptide cleavage site and contains the same dibasic motif between B-chain and C-peptide in INS2. Like INS2 proinsulin, D3Z596 ^25^F-G^79^ is 4.6-fold higher in abundance in β*Cpe*KO. Correspondingly, canonical D-peptide and D-peptide truncation all present with decreases in abundance similar to INS C-peptide proteoforms.

**Figure 7.**
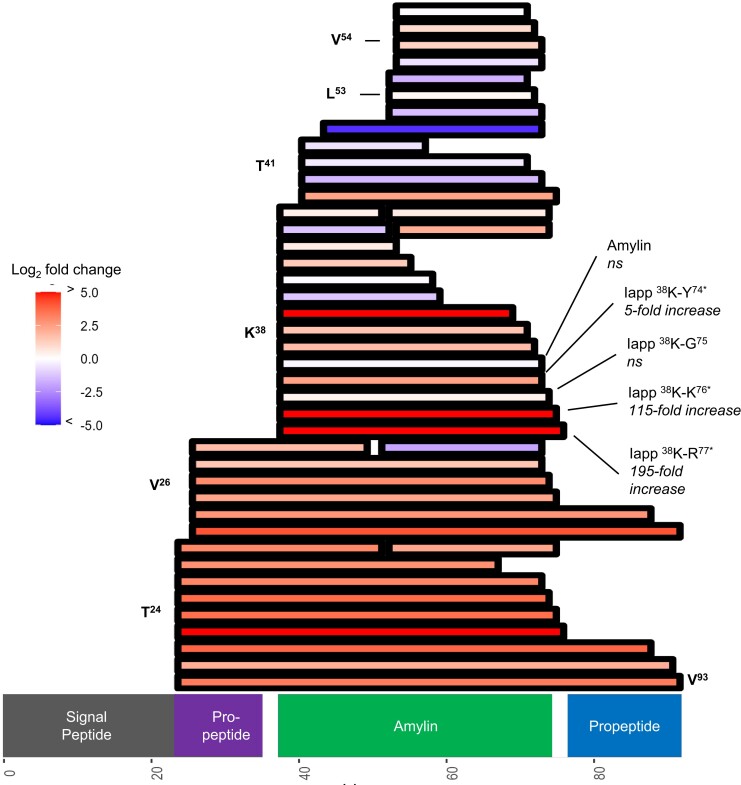
Mapping of IAPP proteoforms with statistically significant and insignificant (adjusted *P* value threshold of .05) changes in abundance (log_2_ fold-change threshold of ±1). Each rectangle indicates a unique proteoform. For proteoforms with text labels, * indicates statistically significant (adjusted *P* value < .05). Proteoforms containing unknown modifications are excluded from this figure.

IAPP (amylin), a related major pancreatic hormone, also displays significant changes with its proteolytic processing due to the loss of CPE. Like insulin, proIAPP is processed into an active form after endoproteolytic cleavage by PCSK1 and PCSK2 followed by dibasic amino acid removal at the C-terminus with CPE (Supplementary Fig. S9 (29)) (35). IAPP also has an amidation step that removes the Gly^75^ residue, leaving the C-terminal Tyr^74^ amidated. Mapping of IAPP proteoforms reveals how processing is impacted, with numerous changes in abundance being observed across different IAPP regions (Fig. 7). Several IAPP proteoforms, containing the propeptide regions extending across the dibasic amino acids, show considerable increases in abundance. The IAPP proteoform ^38^K-R^77^, which contains the C-terminal dibasic residue site cleaved by CPE, has a ∼200-fold increase in abundance—the largest fold-change in abundance among all proteoforms in the entire dataset. The next proteoform in the IAPP/CPE processing pathway, ^38^K-K^76^, shows a 115-fold increase over WT islets. This proteoform is followed by ^38^K-G^75^, which is processed by peptidylglycine α-amidating monooxygenase (PAM). ^38^K-G^75^ is not significantly different in abundance; however, the deamidated intermediate lacking the C-terminal Gly (^38^K-Y^74^) is 5-fold higher. Despite the nearly 200-fold increase in IAPP ^38^K-R^77^ and 115-fold increase in ^38^K-K^76^, the biologically active IAPP proteoform (^38^K-Y[Amide]^74^) is not significantly different between β*Cpe*KO and WT islets (log_2_ fold-change −0.23 and adjusted *P* value of .35). Therefore, as opposed to insulin, compensatory mechanisms appear to enable homeostatic levels of bioactive IAPP in the absence of CPE. Taken together, these results demonstrate the broad changes that occur across multiple pancreatic hormone processing pathways when CPE is conditionally knocked out from mouse islet β-cells.

## Discussion

Despite the recognized role of altered prohormone processing in diabetes, the details of such processing products are still not well understood. Herein, we have presented a comprehensive top-down proteomics analysis of the outcomes of dysfunctional prohormone processing in a β*Cpe*KO mouse model. While certain processing defects were to be expected, the extent and complexity of the changes were substantial. Including the expected functional and incompletely processed forms of insulin, 468 proteoforms of INS1 and INS2 were detected using top-down proteomics across WT and β*Cpe*KO mice, 220 of which could be completely characterized at the molecular level (ie, contained no unknown modifications). Many changes in proteoform abundance between *Cpe* KO and WT mice aligned with the anticipated enzymatic function of CPE (such as in the case with INS1 and INS2 processing). In the case of INS, it was shown that canonical A-chain, B-chain, and C-peptide were all significantly reduced in abundance while incompletely processed proteoforms were correspondingly increased. However, many changes extend beyond the typical exopeptidase cleavage activity that would be expected in a deficient β*Cpe*KO model. For example, INS intermediates (such as proinsulin) that require processing by prohormone convertase enzymes PC1/3 and PC2 were significantly increased in abundance. Additional truncations and alteration were observed, which could be in part due to the increased intracellular concentrations of proteoforms leading to atypical processing and presentation by alternate cellular machinery, degradation mechanisms, or some other means.

CPE is known to act on multiple pancreatic hormones, and the abundances of many of these hormones were also altered as might be anticipated by loss of CPE. Notable examples include IAPP, SCG2, CHGA, SST, and CHGB. IAPP is exemplary in this regard, where elevations greater than 200-fold were observed for some unprocessed proteoforms. Surprisingly, the biologically active IAPP proteoform amylin was unchanged in abundance, which is consistent with our published Western blot data (17). One possible mechanism is the compensatory effect of another exoprotease CPD in cleaving the C-terminal basic residues (36) despite the loss of CPE.

Intracellular accumulation of dysfunctional proteoforms could also induce cellular stress (37) and activate additional corrective and compensatory mechanisms such as autophagy (38). We observed numerous dysregulated proteins and proteoforms that are not classified as canonical CPE substrates. EIF5A is one example, where a hypusinated proteoform was found to be increased in β*Cpe*KO mice. The hypusinated EIF5A is involved in resolving ribosomal stalling and appears to also play a role in islet inflammation (39, 40). These dysregulated proteins extend well outside of known hormone processing functions and include S100A13, which is thought to be an autocrine/paracrine signaling protein, and H2BC4, which is a histone nucleosome subunit (41).

Other notable proteoforms included HMGN3b (Hmgn3 ^30^L-N^77^, decreased 31-fold in β*Cpe*KO), which is an alternative splice isoform derived from *Hmgn3*. An equivalent HMGN3a proteoform (HMGN3 ^30^L-E^99^) was decreased more than 16-fold in β*Cpe*KO islets. The high mobility group nucleosome-binding domain-containing proteins (HMGN) are a family of intrinsically disordered proteins that bind nucleosomes to alter global or local chromatin structure (42). HMGN3 is most strongly expressed in the pancreas and is alternatively spliced into HMGN3a and HMGN3b. *Hmgn3*^−/−^ mice have ∼50% higher insulin in serum along with a ∼50% reduction in GLUT2 protein levels over *Hmgn3*^+/+^ littermates (43, 44). Therefore, the lower abundance of HMGN3 may be part of a feedback mechanism intended to increase insulin secretion to compensate for defects brought on by loss of CPE. Another intriguing example is TPT1, which we observed to have complex posttranslational processing and was increased overall in *Cpe* KO mice. TPT1 is known to be essential for β-cell mass expansion during development as loss of TPT1 in mice results in decreased β-cell growth, reduced β-cell mass, and reduced insulin secretion (45).

The application of quantitative top-down proteoform profiling is still a burgeoning area in proteomics and will have significant impact as it matures (46). Here, we have applied some of the latest advancements in top-down proteomic analysis in order to better understand pancreatic islet hormone processing dysfunction within the context of *Cpe* deletion. The proteoforms we identified may prove useful in diagnosing early signs of β-cell dysfunction in individuals at elevated risk of T1D and can likely be used to provide target pathways for interventional care where this type of dysfunction is observed. Further applications using this top-down proteomic approach on human samples will provide much needed insight into the translatability and applicability of pancreatic hormone proteoforms to T1D.

## Data Availability

Original data generated and analyzed during this study are included in this published article or in the data repositories listed in References. Specifically, supplemental materials including supplemental figures and tables are available at figshare (an online open access repository) (29). Detailed processed data and all raw data files have been deposited into the MassIVE data repository and can be accessed via accession MSV000092097 (47).

## Abbreviations

ABC: ammonium bicarbonate
CPD: carboxypeptidase D
CPE: carboxypeptidase E
CV: compensation voltage
D-peptide: Disjointing peptide
FA: formic acid
FAIMS: high-field asymmetric ion mobility
HMGN: high mobility group nucleosome-binding protein
IAPP: islet amyloid polypeptide
KO: knockout
PC: prohormone convertase
RSD: relative standard deviation
T1D: type 1 diabetes
TPT1: translationally controlled tumor protein
WT: wild-type
